# Smart Containers Schedulers for Microservices Provision in Cloud-Fog-IoT Networks. Challenges and Opportunities

**DOI:** 10.3390/s20061714

**Published:** 2020-03-19

**Authors:** Rocío Pérez de Prado, Sebastián García-Galán, José Enrique Muñoz-Expósito, Adam Marchewka, Nicolás Ruiz-Reyes

**Affiliations:** 1 Telecommunication Engineering Department, University of Jaén, Science and Technology Campus, 23700 Linares (Jaén), Spain; sgalan@ujaen.es (S.G.-G.); jemunoz@ujaen.es (J.E.M.-E.); nicolas@ujaen.es (N.R.-R.); 2 Institute of Telecommunications and Informatics, University of Technology and Life Sciences, Prof. S. Kaliskiego 7, 85-791 Bydgoszcz, Poland; adimar@utp.edu.pl

**Keywords:** fog computing, IoT, cloud computing, soft-computing, machine learning, containers, docker, microservices, intelligent scheduling, cloud service providers

## Abstract

Docker containers are the lightweight-virtualization technology prevailing today for the provision of microservices. This work raises and discusses two main challenges in Docker containers’ scheduling in cloud-fog-internet of things (IoT) networks. First, the convenience to integrate intelligent containers’ schedulers based on soft-computing in the dominant open-source containers’ management platforms: Docker Swarm, Google Kubernetes and Apache Mesos. Secondly, the need for specific intelligent containers’ schedulers for the different interfaces in cloud-fog-IoT networks: cloud-to-fog, fog-to-IoT and cloud-to-fog. The goal of this work is to support the optimal allocation of microservices provided by the main cloud service providers today and used by millions of users worldwide in applications such as smart health, content delivery networks, smart health, etc. Particularly, the improvement is studied in terms of quality of service (QoS) parameters such as latency, load balance, energy consumption and runtime, based on the analysis of previous works and implementations. Moreover, the scientific-technical impact of smart containers’ scheduling in the market is also discussed, showing the possible repercussion of the raised opportunities in the research line.

## 1. Introduction

The adoption of container-based lightweight virtualization solutions is rapidly growing in cloud, fog and IoT networks today [1]. On the one hand, the provision of computational infrastructures, applications and services through containers is a current priority for the most important cloud service providers like Microsoft Azure [2], Amazon Web Services [3] and Google Compute Platform [4]. The main reason resides on the reduction in power and costs in infrastructure, and high execution speeds in the provisioning of microservices achieved in comparison to traditional virtualization technologies such as virtual machines (VMs). On the other hand, containers are considered the first practical virtualization technology of Fog-IoT networks, due to the limited computing resources that their deployment requires compared to the rest of virtualization solutions nowadays [5,6,7,8]. However, their further expansion in cloud, fog and IoT networks critically depends on various precursor conditions, such as the design of more efficient containers’ schedulers. 

A key aspect in containers’ scheduling is the possibility to distribute containers taking into account the dynamic availability and uncertainty in the state of the resources, and the particularities of specific microservices in cloud-fog-IoT networks. This state-aware scheduling could improve the results in terms of runtime, latency, flow-time, power consumption, etc. in comparison to many traditional scheduling strategies currently deployed in dominant containers´ management systems in the market. Particularly, for the management of open-source Docker containers, de facto standard today, there are currently three fundamental tools, all of them also open-source: Docker Swarm [9], Apache Mesos [10] and Google Kubernetes [11]. These tools currently employ classical scheduling strategies, frequently random, static and priority-based, that provide very limited flexibility in the containers´ distribution.

Besides, the performance of containers’ schedulers should be adapted to the different interfaces of cloud-fog-IoT networks and microservices to achieve their highest performance. For instance, it must be noted that the interface cloud-to-fog is generally associated to the reduction of runtime, whereas fog-to-IoT interface is joined to the reduction of latency in many applications. Nevertheless, none of the current smart containers’ scheduling strategies in literature are optimized for specific interfaces of the cloud-fog-IoT networks and type of microservices. Thus, the possibilities of the interplay of these networks to provide integrate services are still not harnessed in all their vast potential.

Thereby, based on the study of previous works both in market implementations and scientific literature in containers’ scheduling in Cloud-Fog-IoT, this work is presented. The contribution of the paper is to raise and discuss two challenges and opportunities in containers’ scheduling and their associated scientific-technical impact:

- First, the absence of intelligent scheduling strategies for containers in the major Docker containers’ management tools today, the open-source solutions Docker Swarm, Apache Mesos and Google Kubernetes, is studied. Specifically, it is discussed how the incorporation of soft-computing-derived strategies such as fuzzy logic (FL), evolutionary computation (EC), and bio-inspired computation strategies, as well as diverse machine learning (ML) strategies such as neural networks (NNs) and derived deep learning (DL), represents an open issue with major advantages [12,13]. To be precise, it can allow millions of users and administrators of these containers’ management tools to achieve a more efficient and personalized scheduling of their microservices, based on their specific objectives and applications in fog, cloud or IoT. Although these techniques have been largely proved effective in the scheduling of tasks and VMs in the last years [14,15,16,17,18], their adaptation and adoption in containers’ scheduling represent multiple challenges as well as opportunities. These challenges and opportunities have scarcely been explored and analyzed at the time of writing and motivates this work [19,20].
- Secondly, the possible benefits of specific smart containers’ schedulers for Docker Swarm, Apache Mesos and Google Kubernetes, for the different interfaces in cloud-fog-IoT networks and type of microservices are analyzed. Specifically, this work is mainly devoted, on the one hand, to suggest the convenience of optimization in the fog-to-cloud and IoT-to-cloud interface of the runtime, and, on the other hand, the optimization of latency, load balance and energy in the fog-to-IoT interface. Hence, it is proposed to consider interface-based scheduling solutions for very frequent objectives in the execution of microservices.
- Thirdly, the scientific-technical impact in the market of the proposed challenges and opportunities is also discussed, in order to show the significance of the research line.

This manuscript is organized as follows. In Section 2 and 3 the fundamentals of cloud-fog-IoT networks, and containers are presented, respectively. Next, previous works in containers’ scheduling in the market and literature are described in Section 4. In Section 5, the challenge of the implementation of smart containers for the main containers’ management platforms today is discussed. Also, in Section 6, the challenge to design specific smart containers’ schedulers for the different cloud-fog-IoT interfaces is studied. Consecutively, Section 7 discusses the scientific and technical impact of the raised open challenges and opportunities, and Section 8 points out future research lines. Finally, the main conclusions of the work are drawn in Section 8.

## 2. Fundamentals of Cloud-Fog-IoT Networks

Fog computing is a terminology introduced by the CISCO company in 2012 [5]. It represents an emerging computing framework designed to meet the challenges of cloud computing or utility grid in relation to latency-sensitive applications [21,22,23,24,25,26,27,28,29,30]. Fog computing is supported by the OpenFog consortium, which authors some relevant white papers in the field such as [31]. The concept of fog comes from the idea of “approaching the cloud closer to the earth”, that is, making it possible a greater proximity of the cloud to the end users. In this way, fog computing constitutes an extension of cloud computing that also considers the processing in external nodes to the cloud system and closer to the end user. In the fog, the execution of some application elements (specially, those latency-sensitive) can be done at the boundaries of the computing network, whereas others (typically computer-intensive and delay-tolerant application elements) can be run on the cloud. Thus, the fog offers advantages like low latency, achieved by processing the workload at the edge of the network in the fog nodes, and short runtimes, by executing compute-intensive applications in the cloud nodes.

**Figure 1:**
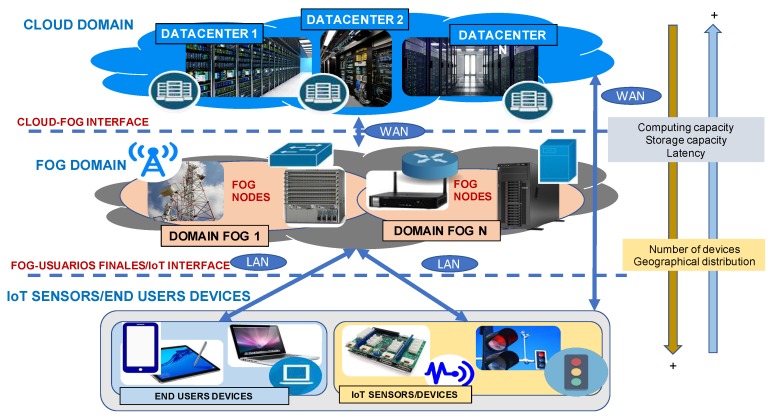
Relationship between fog computing, cloud computing and IoT sensors/end users: cloud-fog-IoT networks.

In the current state of the art [5,32], it is claimed that cloud computing is not suitable or applicable for most of IoT developments, frequently supported by sensor networks. In this context, fog computing has emerged as an opportunity to significantly improve the interplay of these two technologies, and as a consequence, to provide integrated services requiring their cooperation. Nevertheless, it must be highlighted that the field of application of fog computing lies beyond IoT applications, and encompasses areas such as content distribution, of great importance today for companies such as Netflix, HBO or Amazon. Fog computing, however, unlike other related computing paradigms such as mobile ad hoc cloud computing or mobile edge computing, fog computing is closely related to a supporting cloud, that is, it needs the existence of an associated cloud computing network as it cannot perform independently. This justifies why the interactions between the fog and the cloud are of special relevance nowadays [5], and accordingly, the relevance of the improvement of the management and interoperability of these networks. Figure 1 graphically represents the relationship of fog computing and cloud computing and end users or IoT sensors/devices. It must be noted that content centric networking (CCN), information-centric networking (ICN) and name data networking (NDN) represent emerging internet architectures with an information-centric approach at the network level in contrast to host-centric approach of traditional IP-based ones. These approaches can also be deployed with the support of cloud, fog, edge and IoT infrastructures and scheduling and catching strategies are proposed at this level [33,34].

As described in recent surveys [35], multimedia IoT is taking advantage of fog, edge and cloud computing in diverse applications today. This potential is revealed through several current use cases in the context of video on demand (VoD), cyber systems, traffic monitoring and smart vehicles, distributed control of intelligent buildings, smart grid, smart cities, smart health, smart agriculture, real-time multimedia, multimedia in industry, etc. [5,25,26,35,36,37]. In addition, it must be highlighted the importance of fog computing in health care systems. For example, the prediction of falls by patients with stroke [38,39], where fall detection learning algorithms can be dynamically implemented in a fog-based platform. Also, fog computing supports other recent health applications such as the assistance system used by Google Glass equipment to enable those users with low mental acuity to accomplish tasks like pronouncing the names of people they cannot remember [40]. Hence, cloud-fog-IoT networks is revealed as one of the most versatile computational frameworks today.

## 3. Fundamentals of Containers 

### 3.1. The Concept of Container

In order to shorten the differences between the high-demanding computing needs of multiple applications of interest nowadays, and the reduced hardware capabilities of IoT nodes/end users or Fog nodes, a variety of middleware frameworks have been suggested in the last years [6]. For example, small VMs supported by Java and Python runtime environments have been developed to allow sensors and other IoT devices’ programming, as well as code mobility [41,42,43]. It must be underlined that the efficiency of the applications strongly depends on the supporting virtualization technology, which bounds flexibility in hardware and involves limiting dependencies in code. Therefore, recent years have also witnessed an increasing attention to lightweight virtualization solutions, like Docker [44] and LXC [45] containers. These technologies allow a more efficient implementation of virtualized applications/services regarding virtualization technologies based on hypervisors or VMs and, however, they require a lower overhead [1]. This results in their possible application in low capacity devices, such as IoT nodes/end users or fog nodes. Specifically, a container can be defined as a collection of processes isolated from the remaining parts of the system that encapsulates its associated dependencies. Containers do not require a complete guest operating system (OS), which makes them much lighter in comparison to VMs, typically in an order of magnitude. For example, they can boot just in seconds, faster than VMs, and they are designed to demand a reduced set of resources (below 2 GB of RAM), to be scaled to meet needs, if necessary [44].

Hence, lightweight virtualization platforms incorporate relevant advantages, which make them highly interesting in IoT/end-user or fog networks. Some of these advantages are the rapid generation and booting of virtualized instances, the possibility to have a big number of applications simultaneously on the same host (thanks to the small containers’ images), the reduction of overhead costs, while allowing isolation among the different instances being executed on the same host. Furthermore, container-based services do not imply a strong dependence on a given platform, programming languages or specific application domains, so they offer the flexibility to “develop once, deploy everywhere.”

### 3.2. Docker Containers

Docker is the most widespread type of application containers today. Initially, it was based on LinuX Containers (LXC) [45,46,47], but currently running runC, a CLI software to generate and execute containers created by the Open Containers Initiative [48]. Compared to containers in the system level, for example, OpenVZ and LXC, application-oriented containers such as Docker are better suited to microservices frameworks and, in this way, they are gaining momentum in the deployment of the cloud-fog-IoT architectures. Docker containers integrate a file system organized in layers to share the core of the host or host OS, what it is translated in a more efficient performance in the deployment of instances. For example, executing 10 Docker images on the same host, virtually requiring a 1 GB OS each, would not demand 10 GB, unlike VMs, in need of an OS each and so, requiring 10 OS instances.

**Figure 2:**
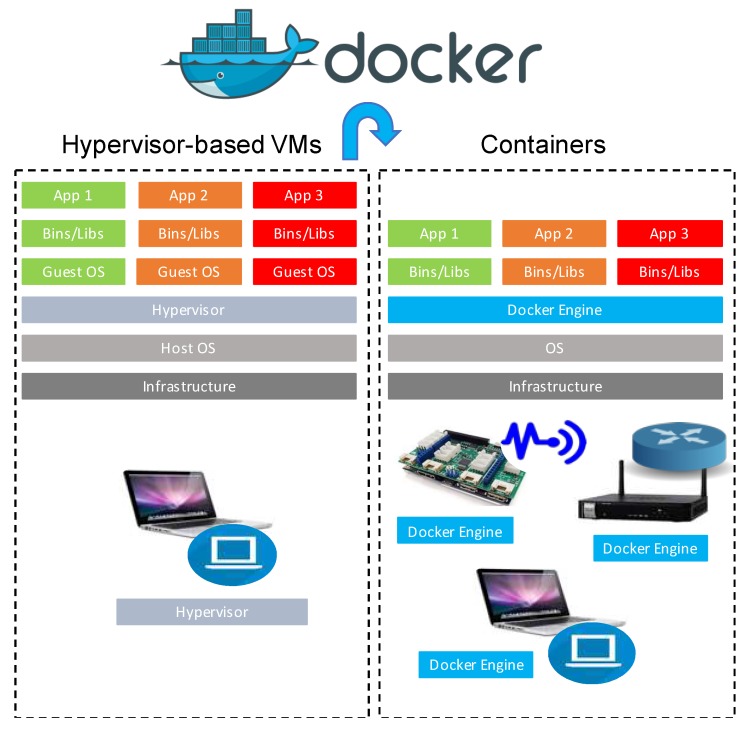
Comparison of Docker containers and hypervisor-based VMs’ layered schemas by Docker Inc.

Docker is founded on images, or more specifically, on snapshots of an OS. In order to generate a new image, it is necessary to start from a base image, conduct the desired changes and save them. The generated images can be shared in public or private records for other developers use, who only need to extract those images. It is very useful to use images to create snapshots of the OS to next use them to generate new containers. The main reason is given by the fact that they are extremely lightweight, as well as easy to use and share, following the Docker’s philosophy [49,50,51]. Containers encompass all the software they need to be executed, to wit, code, system tools, libraries, runtime, etc., and Docker offers a lightweight and stable platform to quickly generate and execute jobs. In particular, Docker’s functionalities are founded on a supporting container engine, Docker Engine, a lightweight containerization architecture integrating all the software tools dedicated to the set up and management of Docker containers. In addition, Docker Engine includes an API allowing the easy build, management and deletion of virtual applications. Figure 2 shows the layered comparison of a Docker container versus a hypervisor-based VM.

## 4. Previous Works in Containers’ Management and Smart Scheduling

### 4.1. Containers’ Management Platforms in the Market

Nowadays containers are often used in cloud computing’s microservice frameworks, where each container can be regarded as a service, communicated and linked to the rest of services by the network. Thereby, these frameworks let every element to be scaled and deployed with independence to the others, but in addition, lightweight container-based virtualization solutions are gaining interest as enablers of a more efficient virtualization technology in IoT/end-user or Fog environments. In fact, the same virtualized container instance can run efficiently on both Fog and IoT/end user nodes, and in the cloud too. Hence, containers can be executed on very limited and relatively inexpensive computing equipment, like Raspberry Pi [6,7,8]. This feature offers a transversal interoperability among different networks, integrating devices with limited resources of the IoT environment/end users or Fog, as well as the cloud high-capable devices. In this way, container-based service provisioning can offer diverse benefits to cloud-fog-IoT networks, allowing applications to be run in a wide set of devices with independence of the underlying hardware. Therefore, containers play today a key role in the enabling technologies for integrated services provision by cloud-fog-IoT networks and their efficient interplay. Nevertheless, in order to maximize the potential of the diverse devices, better solutions are needed that simplify and improve containers’ management.

In containers’ management, the performance of schedulers or brokers, responsible for containers allocation among resources, is especially relevant. The number of used devices and the estimated useful life of the containers can be considered as key factors for a containers’ scheduler to take into account in its decision-making process, in contrast to VMs/tasks scheduler. While the implementation and design of conventional clusters such as Hadoop focuses on the execution of mass jobs [46], containers’ clusters execute hundreds of small instances. A containers’ scheduler in a cluster has multiple objectives: use cluster resources efficiently, work with location restrictions provided by the user, schedule applications quickly so as not to drive them to a waiting state, provide a degree of “impartiality” or balance between resources, be solid against errors, and make the most efficient or convenient allocation in terms of time or power, to name a few. The development of diverse containers’ schedulers for clusters by multiple companies is mainly due to the fact that there is no single solution to solve all problems simultaneously. In this sense, today Google (the main developer of Omega and Kubernetes) opts for an approach that delegates more responsibilities to developers, taking for granted that they will respect the imposed rules related to the priority of their work in the cluster. Meanwhile, Apache (originate organization of YARN) leans towards an approach that imposes capacity, deadlines and equity. The major containers’ management systems are summarized in Table 1 [47].

### 4.2. Docker Containers’ Management Platforms in the Market: Scheduling Strategies

Containers’ schedulers, in general, and Docker containers’ schedulers, in particular, constitute a central component to facilitate the implementation and administration of diverse container-based applications in a series of virtualized or physical hosts. The main scheduling capabilities of the more extended and open-source Docker containers’ management systems today, Docker Swarm, Apache Mesos and Google Kubernetes are analyzed as follows.

• Docker Swarm [9]

Docker Swarm is the containers’ management solution of Docker organization. This containers’ management software, offered by Docker, provides a standard Docker API and its framework consists of two main elements. On the one hand, a machine plays the role of administrator, executes an image of Swarm (a specific Docker image), which is responsible for allocating the containers on other machines called nodes or agents. On the other hand, agents or nodes are machines with a remote Docker API available to the system administrator, once the correct ports are opened when starting the Docker daemon.

In Docker Swarm there are four major scheduling strategies, that is, possibilities for the scheduler to designate a node to execute a container: strategy name or directly node selection, selecting the node with the lower set of containers in execution “spread”, regardless of the load of each container or binpack, selection of the most packaged node (that is, it presents the lower available CPU/RAM), random selection of the node: if the previous strategies select several nodes according to the previous criteria, the scheduler selects a target node among them randomly. The allocation strategy must be determined at the time of setting up the administrator, or the “spread” strategy will be used by default. Particularly, “spread” method is the more extended scheduling strategy in most applications today on a Docker Swarm Mode Cluster, which is a pseudo-random and static scheduling policy.

• Apache Mesos & Mesosphere Marathon [10]

The main aim of Apache Mesos is to provide an efficient and scalable platform supporting a variety of frameworks, currently available or to be developed in the future. Within these frameworks, scheduling systems are considered. Docker container support has been included in Mesos. In particular, the most widespread version of this platform is Mesos with Marathon, which is continuously contributed by Mesosphere, and it provides some possibilities in scheduling such as restrictions, status controls, service discovery and load balancing. The scheduling integrates two major types of nodes: masters and slaves. The master in Apache Mesos transmits the assigned workload to slave nodes and makes new scheduling proposals as others slaves nodes have available CPU/RAM. Hence, slaves execute containers and must inform the master about their free resources. Scheduling policies are based on priority preservation and fairness, and applications can use their own schedulers for workload allocation within this two-level architecture.

• Google Kubernetes [11]

Google Kubernetes is a management tool for Docker containers that uses “tags” and “pods” to run containers [52]. Specifically, the consideration of “pods” constitutes the greatest differentiated factor with regard to the other two management tools, Docker Swarm and Apache Mesos. The “pods” determine group of containers of joint location, which form a global deployed and scheduled service. Google Kubernetes offers a simplified management of clusters of containers compared to an affinity-based scheduling for every container, as is it done in Docker Swarm and Apache Mesos.

The task of the Google Kubernetes’ scheduler is to observe the unallocated “pods” and consider predicates as well as priorities to select the nodes where every “pod” must be executed. The initial set up for these predicates and priorities can be reconfigured with new policies. Kube-scheduler is the standard scheduling tool and it can be executed as part of the control plane. The associated scheduling strategies are founded on a scoring strategy, where the scheduler ranks the feasible nodes and assigns “pods” according to this defined rank. The rank can be stablished statically based on 13 different scoring criteria. Some of the most significant are the selection founded on balanced resource usage (BalanceResourceAllocation policy), predefined preferences (NodeAffinityPriority policy), existence of cached images in nodes (ImageLocalityPriority policy), not assigned nodes (ServiceSpreadingPriority policy) and random spread across hosts (SelectorSpreadPriority policy).

### 4.3. Smart Containers’ Scheduling Strategies in Scientific Literature

Currently just two major works [19,20] can be found in relation to smart containers’ scheduling and their implementations in Docker Swarm, Apache Mesos and Google Kubernetes. On the one hand, in [19] a fuzzy reinforcement learning strategy is proposed for microservices allocation. On the other hand, [20] suggests the consideration of a Swarm Intelligence-based strategy for scheduling in big data applications. In both cases it must be noted that proposals are restricted to smart containers’ scheduling in cloud computing, and suggestions for smart containers’ scheduling in Fog and IoT networks remains unexplored. Also, it is worth mentioning that other works can be found in recent literature related to containers’ scheduling in fog/IoT networks as well as in cloud computing, such as [53,54,55,56,57]. However, these works do not propose specific optimized solutions for every cloud-fog-IoT interface. Furthermore, these locality or network-aware solutions do not take advantage of the possibilities of soft-computing. Hence, this field represents an important research opportunity, as well as a challenge in Docker-supportive cloud-fog-IoT networks. Table 2 summarizes the previous works in advanced containers’ scheduling found in literature and discussed though this study.

In this market and literature context, this work arises two challenges and opportunities in Docker containers’ scheduling for cloud-fog-IoT. The specific discussions are set out in the next sections.

## 5. Challenges and Opportunities. Smart Scheduling in the Dominant Containers‘ Management Systems

### 5.1. Challenge Statement

As it can be inferred from the analysis of previous works, neither Docker Swarm, Apache Mesos nor Google Kubernetes offer the possibility to select different scheduling optimized strategies for latency, execution time and/or power, at the discretion of the network administrator. Just a few reduced sets of static and inflexible strategies are being considered now. In a cloud-fog-IoT scenario, featured by the dynamism and uncertainty in the state of resources and microservices, the role of containers’ scheduling strategies based on soft-computing should be taken into account. Thus, it is proposed to analyze the convenience of using intelligent systems to conform smart containers’ schedulers. These schedulers, on the basis of the current state of the resources and the characteristics of the microservices/applications to be executed, could perform the assignment of containers (or start-up of containers) to hosts in terms of diverse objectives. Particularly, the integration of smart containers’ scheduling in Docker Swarm, Apache Mesos and Google Kubernetes is suggested on the basis of multiple successful previous works in the application of soft-computing techniques such as FL, EC and bio-inspired metaheuristics, ML, etc. in distributed networks for tasks and VMs.

It should be noted that the use of containers is not restricted to cloud or grid networks, so the incorporation of more efficient capabilities for containers managing to Docker Swarm, Apache Mesos and Google Kubernetes should consider the peculiarities and aims of other networks too in their optimization. For example, containers’ scheduling strategies should include latency optimization, which is the fundamental objective of the adoption of fog technology. However, it should also consider other networks’ optimization goals. Hence, for instance, in a cloud network the final execution time is usually a priority, or in mobile edge computing power consumption is critical. The specific objectives would depend on the purpose of the specific cloud-fog-IoT network, such as reducing latency, total runtime, power, or enable load balancing, and dynamical adaption to changing conditions.

### 5.2. Suitable Techniques 

The features of soft-computing techniques and their results in smart scheduling should be discussed to understand their strengths and also, their possible adaptation to containers’ scheduling. soft-computing [12], Artificial intelligence-derived techniques, are intended to operate in environments subject to uncertainty and inaccuracy. Their application can be beneficial in inherently dynamic and full of uncertainty networks, such as cloud, fog, IoT, edge mobile computing, etc., where, as in the case of grid systems no quality of service (QoS) can be provided without taking into account a “known” state of the system [58]. This study proposes the application of various soft-computing techniques for the improvement of containers’ schedulers for the provision of microservices of current interest in cloud, fog, IoT, edge mobile computing networks, etc.

Specifically, founded on previous works in smart scheduling, the application of FL and EC, NNs and derived DL, and probabilistic reasoning (PR) approaches, and associated knowledge acquisition strategies based on ML, is to be highlighted [12,13]. These soft-computing strategies differ from other known as hard-computing strategies in their greater flexibility to dynamism and adaptation through learning, as well as the interpretability of their solutions in some cases. Indeed, beyond their learning capabilities and adaptation to dynamism, a key aspect in their success can be found in the reduction in the computational requirements from traditional proposals. Moreover, this computational effort is unnecessary in problems where a loss in accuracy in the final solution’s precision can be sacrificed in order to reduce costs and increase simplicity. Additionally, these strategies are able to model vague, imprecise or noisy information, which is inherently present in the state of workload and resources in many scenarios in real applications, that cannot be handled by hard-computing strategies. Hence, soft-computing approaches offer a possible solution to problems, where necessary data is not completely available or featured, or presents imprecision or noise by its own nature. This is the case for the state of resources and workload in many distributed networks such as cloud, fog and IoT networks.

Particularly, when managing systems with uncertain state/data and with high dynamism the suitability of the application of FL has to be specially remarked [13]. FL is basically founded on the principle that the human reasoning is approximate by nature, and it suggests a technique for the characterization of this imprecise information in scenarios where vagueness is inevitable, as is generally the case of scheduling in cloud-fog-IoT networks. Multiple previous works can be found in literature using FL to perform an allocation strategy able to considering inherent imprecisions. For example, the recent works of Farid et al. [14], Wang et al. [59] and Ragmani et al. [60] propose diverse FL-derived strategies for tasks/VMs scheduling in cloud computing. Also, in the field of cloud-fog-IoT, it is to be remarked the work of Mallikarjuna [15], which suggests a scheduling approach for resource management based of FL. Also, the consideration of FL in tasks/VMs scheduling can be found in the work of Zhou et al. [61], where FL strategies are applied in order to achieve an adaptive FL smart broker in grid computing, using the FL control technology to sort out the most suitable computing resource in the network. Also, a fuzzy neural network (FNN) was proposed by Yu et al. [62] to achieve a high-performance scheduling strategy. The strategy considers FL to test the network state related to load, to next consider the NNs to tune the fuzzy sets in automatically. Additionally, Hao et al. [63] suggested a resource allocation strategy based on NNs to achieve the required QoS. Specifically, the selection of computing resources is constrained by QoS criteria and it is done considering NNs. Moreover, the role of EC in tasks/VMs scheduling in diverse distributed systems is to be underlined. Of special relevance is its consideration in cloud computing, as it can be appreciated in recent works based on particle swarm optimization (PSO) [64,65], gravitational search algorithm (GSA) [16] and hybrid elephant herding optimization [66]. Additionally, some tasks/VM approaches founded on DL are also emerging, such as [17,67].

Besides, fuzzy rule-based systems (FRBSs) represent an extension of traditional rule-based systems that try to join the hard-computing’s accuracy and the soft-computing’s interpretability and flexibility. FRBSs correspond to knowledge-based systems founded on FL and rule-based systems. FRBSs use FL for the characterization of system’s variables and base their decisions on “IF-THEN” rules that represent blurred statements relating these variables. They can provide decisions on complex applications where there is low certainty, through a process of approximate reasoning with characteristics similar to human. Thus, one of the greatest advantages of FRBSs is given by their capacity to deal with vagueness in data and states found in highly dynamic scenarios. These systems have been applied with good results in numerous problems of fuzzy modeling, control and classification [68,69]. Given their features, FRBSs are also increasingly calling the scheduling’ researchers attention for their application in diverse distributed networks [18,70,71,72].

Particularly, the role of FRBSs in smart scheduling must be underlined on the basis of the authors’ own previous experience in the field [73,74,75]. The approximate reasoning of FRBSs in scheduling has been proved advantageous in diverse scenarios in comparison to non-expert strategies. The objective of containers’ scheduling is to select that host to execute the containerized applications/microservices at each stage of the allocation process, which can improve the efficiency of the cloud, fog, IoT, etc., and thus, the QoS provided by these networks to the users. It is suggested to make this decision taking into account that the information of the state of the cloud-fog-IoT domains has a degree of imprecision, given the high dynamism of these networks. Furthermore, FRBSs schedulers’ knowledge can be interpreted to understand the adopted decisions in scheduling, and learn about the network where they take these decisions. Hence, the previous application of these intelligent techniques in grid/cloud systems for computational tasks and VMs drives now to its consideration in Docker-enabled cloud-fog-IoT networks. Specifically, the integration of these systems in the most widespread container management solutions today, Docker Swarm [9], Apache Mesos [10] and Google Kubernetes [11] could bring many improvements in QoS as proved in related scenarios.

Associated to this aim, it is also necessary to point out that the performance of expert systems is strongly related to the quality of their knowledge bases (KBs). KBs describe the rules and representation of the variables supporting the expert system’s decisions [12]. Due to the changing nature and complex search spaces typical of computer networks such as Fog, automatically obtaining a high-quality knowledge base is a complex process. As found in current literature, a general trend is to apply a collection of learning strategies to obtain rule bases (RBs) derived from EC. Particularly, both the adaptation and modification of classic learning strategies in FRBSs, as well as new strategies that offer improvements in terms of quality of knowledge, computational effort and speed, such strategies based on genetic algorithms (GA), differential evolution (DE), and strategies derived from swarm intelligence (SI) with particle swarm optimization, can be found in previous works [76,77,78,79,80]. In addition, given the diverse and even contradictory nature of the criteria to be optimized for every state of resources and microservices, knowledge acquisition in smart schedulers through multi-objective techniques could also be beneficial [76]. Thereby, it could also be convenient to apply multi-objective knowledge acquisition strategies, typically based on the general Pareto theory, to simultaneously improve latency, runtime, balance and/or power consumption, etc., some of the more common QoS parameters in cloud-fog-IoT networks.

### 5.3. Scheduling General Framework

It is also important to analyze the general framework required to adapt the discussed suitable techniques to Docker Swarm, Apache Mesos and Google Kubernetes. The fundamental framework required for the adaptation of intelligent strategies in these platforms can be directly found in their middleware, which enables the straight integration. In each containers’ scheduling solution offered by Docker Swarm, Apache Mesos and Google Kubernetes, two types of logical entities could be adapted to perform the smart scheduling strategies. First, the administrative nodes, which could perform the functions of containers’ brokers. Secondly, the work nodes, which could receive and execute the containerized applications/microservices. In addition, the work nodes could notify the current status of the assigned applications/microservices and their own state, so that the scheduler can perform an intelligent scheduling. As discussed above, soft-computing-derived scheduling strategies or smart scheduling brokers are knowledge-based systems and thus, they require the consideration of information retrieved from the network and microservices dynamically. Note that depending on the specific platform, the name of the work and administrative nodes can change (slave and master nodes in Apache Mesos, for example). However, the philosophy in all platforms is analogous, and so, the adaptation of smart schedulers can also be done parallelly in these three dominant platforms today.

### 5.4. Expected Results

**Figure 3:**
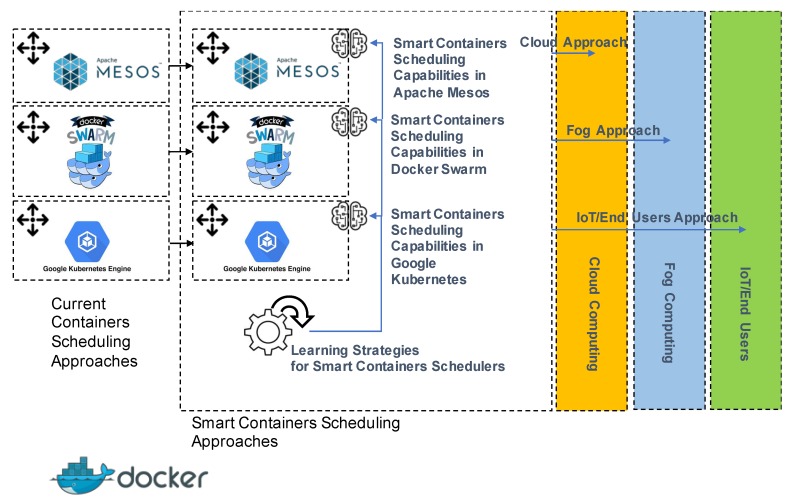
Conceptual schema of the first raised challenge: smart containers’ scheduling in the dominant containers’ management platform. Integration of soft-computing derived strategies for allocation of containers, machine learning supporting knowledge based-brokers and particular design for every Docker-enabled distributed network, such as cloud, fog and IoT infrastructures.

As a result of this research, Docker Swarm, Apache Mesos and Google Kubernetes would have smart scheduling capabilities. Hence, the network administrators could choose the fundamental characteristics of the expert system, such as the environmental variables and their featuring, to optimize latency, load balance, execution time and/or power, etc. In addition, the network administrator would have the possibility to carry out learning processes to improve the performance of expert knowledge in the advent of significant changes in the network’s operating pattern (due to resources and microservices’ state), which may lower the efficiency of the expert knowledge. Nevertheless, it should be again pointed out that these capabilities depend on the efficient incorporation of expert scheduling capabilities, but also, on the good adaption and integration of learning capabilities. Figure 3 graphically depicts the raised challenges in smart containers’ scheduling in the dominant containers’ management platforms 

## 6. Challenges and Opportunities. Smart Containers’ Scheduling in the Cloud-Fog-IoT Interfaces

### 6.1. Challenge Statement

The second main challenge raised in this work is the need for containers’ scheduling strategies adapted to the three general interfaces in cloud-fog-IoT networks. In particular, containers’ scheduling strategies in Docker Swarm, Apache Mesos and Google Kubernetes are currently generically used in the different levels of the cloud-fog-IoT networks. Furthermore, no distinction is currently be done for the scheduling in the different levels in the state of the art at the time of writing, in spite of the diverse goals that are pursued in the interplay between these Docker-enabled networks. Hence, derived from the previous works’ analysis in Section 4, it can not only be inferred that the consideration of smart containers’ schedulers in literature and major containers management systems in market is practically inexistent. Also, no advanced strategies are suggested for the different interfaces in cloud-fog-IoT. In this section, the novelty and advantages of the incorporation of optimized smart containers’ scheduling for the diverse interfaces are brought to light.

### 6.2. Interfaces in Cloud-Fog-IoT Networks

The structure of cloud-fog-IoT networks define the different interfaces to be analyzed. Figure 1 depicts the relationship of fog computing, cloud computing and IoT/end users’ and the essential connectivity among these networks, as introduced in Section 2. It can be observed that cloud-fog-IoT networks consist of a single or multiple cloud, fog and IoT domains, monitored and managed by the same or diverse providers. In turn, each cloud, fog and IoT domain include multiple nodes that may integrate sensors, switches, routers, gateways, access points, computers, smartphones, decoders, etc. Particularly, in regard of the IoT/end users’ level, it can be appreciated that it consists of two main domains or levels. The first one including the end-user systems, and the second one encompassing the IoT devices typically involving multiple sensors networks. It should be noted that one of these two domains, the IoT level or the end users’ level, may is not present in the cloud-fog-IoT networks’ topology. This is, for example, the case of cloud-fog-IoT networks’ architectures for content distribution or content delivery networks (DCNs) or cloud content delivery networks (CDCNs), where there is no IoT level involved. On the other hand, as it can be observed in Figure 1, communication among the IoT level/end users and the fog level is typically achieved through a local area network or LAN. On the other hand, the connection between the IoT/end users’ level and the cloud level frequently requires a wide area network or WAN, whether or not through the Fog level. Therefore, in a cloud-fog-IoT network the following three scheduling interfaces can overtly be distinguished:

- Cloud-to-fog
- Fog-to-IoT/end users
- Cloud-to-IoT/end users

For each of these interfaces, specific designs of containers’ scheduling cannot be currently found in literature or market. However, it can be easily inferred that optimized strategies for each specific interface could be significantly beneficial. It must be noted that it is difficult, or infrequent, to be able to design scheduling strategies providing the highest efficiency in latency, runtime, flowtime, load balance and/or power consumption, simultaneously. Notwithstanding, each of the introduced interfaces generally only need to perform in pursue of the optimization of a limited set of these parameters, associated to the goal of the interplay of the two involved networks and type of microservice. For instance, in the case of cloud-to-fog interfaces, fog networks essentially have their origin in reducing the latency of cloud networks, and suggest an interplay between them to achieve this objective, while still being able to offer users reduced runtime. Therefore, the optimization of these parameters is of special interest to all those network administrators who adopt fog-cloud networks as an alternative to independent cloud networks. In particular, Docker Swarm, Apache Mesos and Google Kubernetes, as discussed in the previous section, do not offer advanced containers’ schedulers neither in terms of diverse criteria on the basis of resources state and application type, nor in optimized solutions in terms of interfaces.

### 6.3. Scheduling General Framework

In this context, the design and implementation within Docker Swarm, Apache Mesos and Google Kubernetes of smart schedulers, specifically optimized for the concrete particularities and needs of each of cloud-fog-IoT/end user’ interface, is also raised as an open issue. To achieve this goal, it would be necessary to study the most convenient input variables or features for the expert system in each interface, such as the characteristics of the involved networks in the interface, the number of containers and applications/microservices to be considered, the characteristics of the applications/microservices, the suitability of load balancing due to device power limitations, etc. 

Furthermore, for each interface the location of the administrative nodes and the working nodes must be defined. In the case of the cloud-to-fog interface, an administrator node should be used in the fog network as broker. In this way, depending on the particularities of the applications/microservices, such as the latency and execution time requirements, the broker node can decide on scheduling in the nearby nodes to generate low delays or sending applications/microservices to the cloud network, if latency is not critical for any part of the service. This type of infrastructure is of particular importance, for example, in intelligent video surveillance systems. In these systems, on the one hand, applications/microservices related to the detection of events are destined to nearby nodes that can meet the criteria of latency. On the other hand, heavy workload related to the identification of events are delegated to the cloud network, which can execute the containerized applications in an optimized way in terms of runtime.

A similar schema could be also considered for the cloud-IoT/end users’ interface, bearing in mind the also latency-tolerant applications typically involved. On the other hand, in the Fog-IoT and fog-end user interface, some nodes within the Fog network could be used as administrators or brokers of containerized applications/microservices, and the rest of Fog nodes and IoT nodes or end users, as working nodes. Thus, for example, this architecture is also relevant in video surveillance networks, but for cloudless interfaces. In this scenario, sensor networks and devices with low computing capabilities, some belonging to the fog network and others to the IoT network or end user, can be coordinated through a broker node to execute workload with low latency needs, such as the detection of an event. In this case, optimization based on power consumption and load balance could be of special significance, given the nature of the devices in this interface.

### 6.4. Expected Results

**Figure 4:**
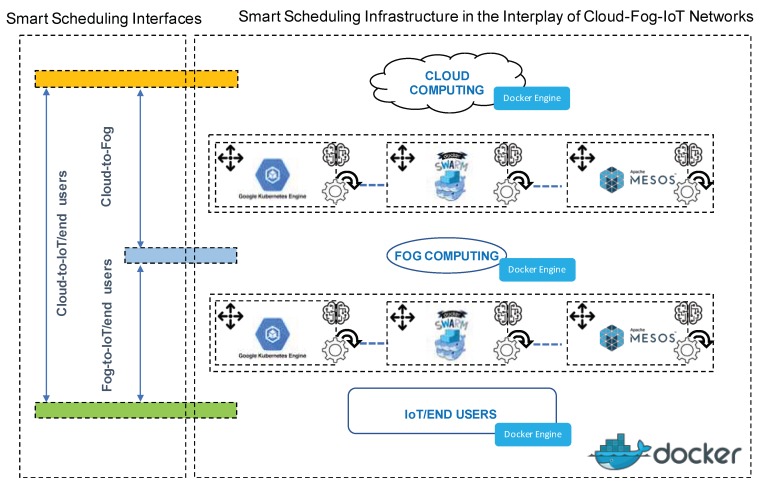
Conceptual schema of the first raised challenge: smart containers’ scheduling in the diverse cloud-fog-IoT interfaces. Integration of soft-computing-derived strategies for allocation of containers, machine learning supporting knowledge based-brokers and particular design for every Docker-enabled distributed network, such as cloud, fog and IoT infrastructures and interface and/or general sort of application.

As a result of this research, joint to the previous challenge’s results, Docker Swarm, Apache Mesos and Google Kubernetes could integrate smart containers schedulers, each optimized in latency, runtime, load balance and/or power consumption, etc., depending on the concrete interface and/or sort of application, replacing generic and static strategies for all interfaces and/or general application sort currently being used these platforms. Particularly, on the basis of prototypical applications and reason to be of the cloud, fog and IoT networks, two main specific opportunities can be underlined. Firstly, the design and implementation of smart application/microservice schedulers for the cloud-to-fog interfaces and cloud-to-IoT with optimization of runtime and latency restrictions. Secondly, design and implementation of expert application/microservice schedulers for the fog-to-IoT/end users’ interface with latency optimization, power consumption and/or load balancing, simultaneously. Finally, Figure 4 shows the conceptual schema for the second raised and analyzed challenge.

## 7. Discussion on the Scientific-Technical Impact of the Raised Challenges and Opportunities

As analyzed in the previous section, this work aims to bring to light the advantageous potential improvements in Docker containerized applications/microservices scheduling. Specifically, it is raised the challenge of the provision scheduling for cloud-fog-IoT interfaces/sort of application through intelligent techniques, mainly derived from soft-computing, which have been successfully tested in the scheduling of tasks and VMs in grid/cloud computing. It must be underlined again that the possibility of containers’ scheduling using smart systems is not present in any of the most widespread containers’ management systems, like Docker Swarm, Apache Mesos and Google Kubernetes, and reduced and restricted to cloud systems in the state of art at the time of writing. However, its use would have a scientific and technical impact, as well as derived economic impact, on three main areas which currently employ Docker containers through these management systems: cloud computing, fog computing and IoT networks.

First, in relation to the scientific-technical impact of the research, it should be noted that Docker has been established as the *de facto* standard in containers. Although there are some competing products, they are far behind Docker’s popularity and market growth. Particularly, the announcement in 2016 of Microsoft in relation to its support in both Windows 10 and Windows Server 2016, favored the consolidation and market dominance of these containers. In this way, on the one hand, Docker containers are rapidly being implemented as an alternative to the classic VMs in cloud computing. The reasons can be found in the reduction in power and costs in hardware infrastructure they entail, in addition to portability across platforms, reduced overheads and speed of execution. Thus, its adoption for multiple applications is currently a priority for providers of cloud infrastructures, platforms and services [81], such as Amazon Web Services [3], Microsoft Azure [2] and Google Cloud Platform [4]. In addition, the fact that Docker provides open-source software further increases its consideration by many application developers, estimating that there is currently a 40% annual increase in its adoption. Therefore, an improvement in scheduling in the most widespread containers’ management systems, as proposed in this work for Docker Swarm, Apache Mesos and Google Kubernetes, can result on many benefits. On the one hand, in improved execution efficiency of applications of millions of users of cloud infrastructures. On the other hand, in the promotion of container technologies that generate savings for cloud providers, due to the reduction of associated hardware and power costs to carry out a same service.

Secondly, it is necessary to highlight the impulse that Docker containers represent to make possible the containerization in the fog and IoT expanding networks. Docker containers are considered, in general, as the first practical virtualization technology for Fog and IoT networks, although there are still many efforts to be made to achieve an extensive and efficient deployment (in terms of latency, load balancing, power, etc.) of these containers in the devices of these networks, as is to achieve a more efficient scheduling than currently given by Docker Swarm, Apache Mesos and Google Kubernetes. Thereby, the possible improvements given by the proposed research line can also be understood within the global impact and applications of the fog and IoT networks. In this regard, it should be noted that the fog computing market is estimated to exceed 18.2 billion dollars by 2022 [31]. Table 3 shows the expected evolution of the fog market between 2019 and 2022. However, it should also be noted that this growth is considered dependent on a series of precursor conditions allowing associated technology to emerge completely, including hardware capable of integrating containers without harming the primary functionality of the devices, efficient management of Fog nodes, improvement of security services and administration, as well as the appearance of Fog service providers (fog as a service/FaaS). Moreover, it should be noted that IoT networks, which aims to integrate all types of electronic devices of users, such as medical equipment, cameras and all kinds of sensors, on the Internet for the deployment of smart cities, infrastructure and other services that improve the quality of life, also has a high current importance. In this sense, by 2025, it has been estimated that IoT industry can have an economic impact of $11 billion per year, what corresponds to about 11% of the current world economy, with 1 billion IoT devices distributed worldwide. Since fog computing allows decentralized and knowledge-based processing of huge data volumes generated by the deployed IoT sensors, the improvement in the management of these networks can bring many benefits for society, enabling, for instance, intelligent applications for health care, as discussed in the background of this work, and in general, allowing IoT to reach its vast potential through its cooperation.

## 8. Future Directions

In spite of the wide expansion and maturity of most containers’ management systems, the design and development of smart containers’ scheduling is just in its initial. There are four aspects that are raised in relation to new further research challenges and opportunities:

- Kubernetes, Apache Mesos and Docker Swarm are containers’ management systems that focus on the allocation of Docker containers with fine-grained resource granularity. However, there are other open-source platforms such as YARN where a coarse-grained resource granularity is considered for Docker containers’ scheduling. In these coarse-grained-oriented platforms, the consideration of smart state-aware scheduling solutions could also improve QoS in terms of execution time, latency, flow-time, power consumption, etc., in cloud-fog-IoT applications.
- Resources and microservices’ state consideration in smart scheduling can favor the consecution of a greater QoS. Nevertheless, beyond this purpose, it could be beneficial to consider factors from the administration perspective. This is the case of costs of resources, prices and heterogeneities of services. Involving these factors in the scheduling would imply that a cloud-fog-IoT infrastructure could assess the expenses of microservices’ execution, and adjust to budgets, restrictions or peaks of QoS requirements, with time automatically.
- Failure management is a key procedure in any containers’ management strategy. The consideration of the peculiarities and state of nearby cloud-fog-IoT nodes in the scheduling could be beneficial to support container migration at the event of a failure. Hence, smart containers’ schedulers could integrate failure-focused parameters, that could provide migration-aware decisions.
- It could be relevant to analyze the possibility to incorporate, parallelly to containers’ scheduling, capabilities for autoscaling of resources. In this way, based on the current state of the resources and the type of service or budgets, an intelligent system could automatically scale resources to modify the maximum achievable QoS by the scheduling strategy.

## 9. Conclusions

In a context where containers, in general, and Docker containers, in particular, are the lightweight virtualization technology that has been imposed as the most extended solution for the provision of microservices in cloud, fog and IoT, all the possible improvements in their management have a strong scientific, as well as technological and, therefore, economic impact in markets in full expansion today. This work aims to analyze and bring to light the improvable scheduling systems that are currently used as container scheduling methods in the most widespread containers’ management systems, such as Docker Swarm, Apache Mesos and Google Kubernetes in terms of efficiency in runtime, power consumption, latency, load balance, etc. Moreover, considering previous works in scheduling VMs and tasks in distributed computer networks and their successful results, it is intended to draw the attention of the scientific community to the convenience of the application of intelligent strategies derived from soft-computing. Specifically, two fundamental challenges and opportunities, with limited or no current works available in literature, despite the associated scientific and technical impact, have been raised. First, the incorporation of expert scheduling strategies and learning systems for containers’ scheduling, and particularly, for containers’ scheduling in Docker Swarm, Google Kubernetes and Apache Mesos, considering the specific purposes of the distributed network to be managed. Second, the design and implementation of optimized schedulers based on the different interfaces in cloud-fog-IoT networks regarding the demanded QoS in terms latency, power consumption and/or load balance associated to the interplay of the networks. Hence, this work represents another step to the achievement of advanced global virtualization technologies and the effective interplay in cloud-fog-IoT networks in the search for the efficient provision of microservices.

## Figures and Tables

**Table 1:** Main containers’ management tools compared features.

Main Containers’ Management Tools Compared Features						
Tool	Original Organization	Sharing	Containers Type	Resource Granularity	IP Per Container	Workload
Apollo	Microsoft	No open-source	N/S	Fine-grained	N/S	Batch jobs
Aurora	Twitter	Open-source	Mesos, Docker	Fine-grained	Yes	Long running and cron jobs
Borg	Google	No open-source	Linux cgroups-based	Fine-grained	No	All
Fuxi	Alibaba	No open-source	Linux cgroups-based	Bundle	N/S	Batch jobs
Kubernetes	Google	Open-source	Docker, rkt, CRI API implementations, OCI-compliant runtimes	Fine-grained	Yes	All
Mesos/ Marathon	UC Berkeley/ Mesosphere	Open-source	Mesos, Docker	Fine-grained	Yes	All/Long running jobs
Omega	Google	No open-source	N/S	Fine-grained	N/S	All
Swarm	Docker	Open-source	Docker	Fine-grained	Yes	Long running jobs
YARN	Apache	Open-source	Linux cgroups-based, Docker	Coarse-grained	No	Batch jobs

**Table 2:** Previous works in advanced containers’ scheduling in cloud-fog-IoT networks and main features.

Previous Works in Advanced Containers’ Scheduling in Cloud-Fog-IoT Networks							
Work	Soft-Computing -Based	Cloud	Fog	IoT	Cloud-Fog Interface	Fog-IoT Interface	Cloud-IoT Interface
Joseph et al., 2019 [19]	Yes	Yes	No	No	No	No	No
Liu et al., 2020 [20]	Yes	Yes	No	No	No	No	No
Santos et al., 2019 [53]	No	No	Yes	No	No	No	No
Santos et al., 2019 [54]	No	No	Yes	No	No	No	No
Babu et al., 2020 [55]	No	Yes	No	No	No	No	No
Hong et al., 2018 [56]	No	No	Yes	No	No	No	No
Santoro et al., 2017 [57]	No	No	Yes	Yes	No	No	No

**Table 3:** Fog computing market estimated economic evolution between 2019 and 2022 worldwide in diverse sectors [31].

Amount Per Year and Activity (Thousands of Millions of Dollars)		
Sector within Fog Computing	2019	2022
Wearables	158	778
Smart Homes	99	413
Smart Cities	128	629
Smart Buildings	160	693
Agriculture	362	2118
Utilities	851	3840
Retail Sales	178	509
Data Centers	162	856
Healthcare	503	2737
Industry	524	2305
Transport	582	3296
**Total Activity**	**3707**	**18,174**
